# Isolated perineal plaque as the initial presentation of pemphigus vulgaris

**DOI:** 10.1016/j.jdcr.2025.10.034

**Published:** 2025-10-28

**Authors:** Aster Workineh, Gauri Panse, Mary Tomayko, Alicia J. Little

**Affiliations:** a Yale School of Medicine, New Haven, Connecticut; b Department of Dermatology, Yale School of Medicine, New Haven, Connecticut

**Keywords:** desmoglein 3, direct immunofluorescence, indirect immunofluorescence, isolated pemphigus, localized pemphigus, mucosal-dominant pemphigus, pemphigus, pemphigus vulgaris, perineal plaque

## Introduction

Pemphigus vulgaris (PV) is a rare blistering disorder caused by IgG autoantibodies targeting keratinocyte adhesion proteins, desmogleins.^1^ Most patients present with oral eroded plaques or widespread disease. However, atypical presentations, such as an isolated plaque on the scalp, face, extremities,^2^ or localized oral findings,^1^ can mimic benign dermatoses and delay diagnosis. Although anogenital involvement occurs, it is usually seen in established PV or generalized disease.^3^^,^^4^ Untreated, PV can progress to extensive erosions with significant morbidity and mortality.^1^ We present a case of PV confined to the perineum for over 12 months before progressing to widespread disease.

## Case report

**Fig 1:**
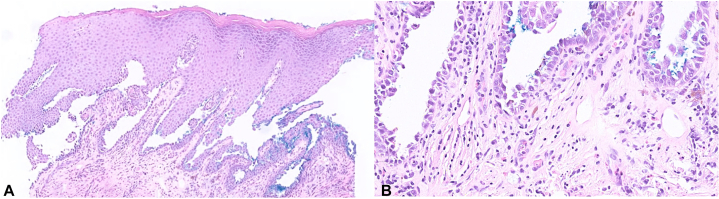
**A,** Skin biopsy demonstrating suprabasal split with acantholysis. **B,** Suprabasal acantholysis with basal keratinocytes resembling a “row of tombstones” and a mixed inflammatory infiltrate in the dermis containing eosinophils. (**A** and **B,** Hematoxylin-eosin stain; original magnifications: **A,** ×200; **B,** ×400.)

An 82-year-old woman with remote history of breast and uterine cancers, type 2 diabetes mellitus and cerebellar ataxia with neuropathy and vestibular areflexia syndrome presented to dermatology with a 9-month history of intermittent perineal irritation. Four months prior, gynecology examination documented perineal fissuring, and skin biopsy demonstrated acantholysis (Fig 1, *A*, *B*). She used betamethasone 0.05% ointment for 1 month, then 3 months of intermittent hydrocortisone 2.5% ointment with some improvement.

**Fig 2:**
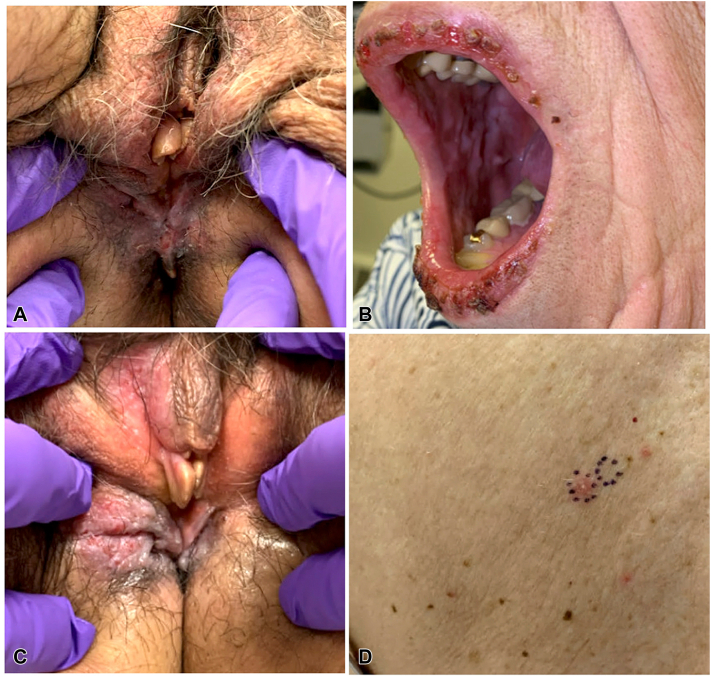
**A,** Photo showing perineal eroded plaque at initial visit (month 9). **B,** Photo showing hemorrhagic crusting of the lips and erosion plaques of the buccal mucosa (month 15). **C,** Photo showing perineal eroded plaque with involvement of the labia majora and interlabial sulcus (month 15). **D,** Photo showing eroded papules lesions of the back (month 15).

On initial dermatology assessment, examination revealed a solitary hypopigmented eroded perineal plaque (Fig 2, *A*) and mild clitoral hood edema; the remainder of complete mucocutaneous examination was unremarkable. Vulvar swabs were negative for Candida or fungi. Differential diagnosis included papular acantholytic dyskeratosis, autoimmune blistering dermatoses, and other less likely acantholytic disorders. Treatment with topical hydrocortisone 2.5% ointment was continued while workup for pemphigus was initiated.

Serum indirect immunofluorescence (IIF) on monkey esophagus substrate was negative. Enzyme-linked immunosorbent assay (ELISA) demonstrated elevated anti-desmoglein-3 antibodies (anti-Dsg3) (91 U/mL; reference: negative < 20 U/mL) and borderline anti-Dsg1 (14 U/mL, negative < 14 U/mL). The patient declined repeat biopsy for direct immunofluorescence (DIF) given stable localized disease.

Three months later, symptoms had improved, and examination showed a stable eroded perineal plaque and minimal erythema of the labia majora; the remainder of complete skin and mucosal examination remained unremarkable.

Six months after dermatology presentation (15 months from initial symptoms), the patient experienced new widespread mucosal and cutaneous erosions and reported persistent hoarseness from suspected laryngitis, initially ascribed to viral upper respiratory infection. Exam revealed multiple hemorrhagic crusted erosions on the lips and hard palate with white plaques on the buccal mucosa (Fig 2, *B*). Vulvar examination revealed pink-white shallow eroded plaques on the labia majora, interlabial sulcus, and perineum (Fig 2, *C*). Few scattered pink eroded papules were present on the back (Fig 2, *D*) and legs. Bacterial culture from the lips grew methicillin-sensitive *Staphylococcus aureus*; herpes simplex virus polymerase chain reaction and fungal cultures were negative.

**Fig 3:**
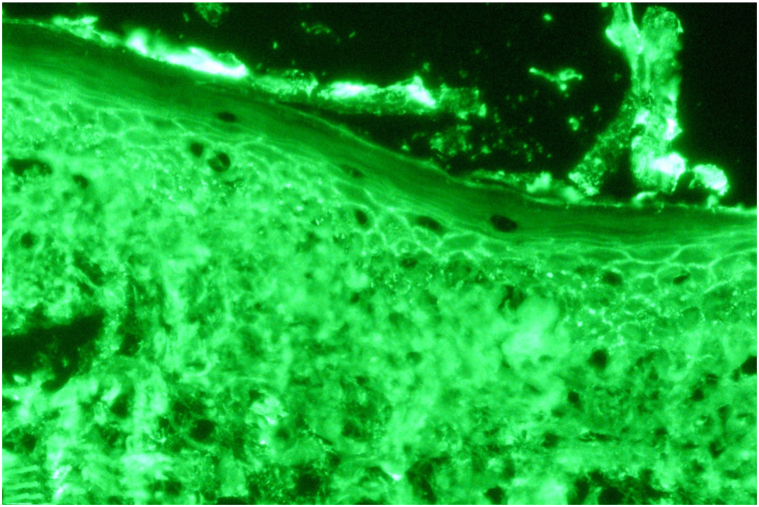
Direct immunofluorescence of perilesional skin, demonstrating net such as, intercellular deposition of IgG throughout the epidermis, consistent with pemphigus vulgaris. (Original magnification: ×400.)

Biopsy of pink papule on the back demonstrated suprabasal acantholysis, and perilesional DIF confirmed net-like intercellular IgG deposition (Fig 3), establishing a diagnosis of PV. Medication timeline review identified no exposures to established PV-inducing drugs; making a drug induced PV etiology unlikely. Repeat serology revealed markedly elevated anti-Dsg3 (1508 relative units [RU]/mL, negative < 20 RU/mL) and negative anti-Dsg1 (19 RU/mL, negative < 20 RU/mL).

Treatment included topical clobetasol 0.05% ointment and dexamethasone 0.5 mg/5 mL swish-and-spit (with clotrimazole for antifungal prophylaxis) for symptom control, and 7-day course of cephalexin for secondary infection. Rituximab was selected because of its efficacy in remission induction and favorable safety profile for patient’s age and history of prior malignancy, type 2 diabetes mellitus, and polypharmacy risk compared with systemic corticosteroids or steroid-sparing immunosupressants.^1^

## Discussion

This case demonstrates that a prolonged minimally symptomatic perineal plaque presentation should prompt consideration of PV. Given chronicity of an isolated lesion and biopsy-proven acantholysis, the initial differential included papular acantholytic dyskeratosis. Papular acantholytic dyskeratosis is a benign, anogenital-restricted disorder predominantly in women and presents as discrete pink or skin-colored asymptomatic, nonprogressive papules or thin plaques.^5^

Other inherited and acquired acantholytic dermatoses considered were Darier disease, Hailey-Hailey disease, and Grover disease. Darier disease usually presents with multiple papules in a seborrheic distribution and nail changes; Hailey-Hailey disease produces grouped erosions in intertriginous areas; and Grover disease causes pruritic papules on the trunk.^6^^,^^7^ None of these features were observed, making these diagnoses unlikely. Pemphigus was another key consideration among acantholytic dermatoses.

Desmoglein expression correlates with pemphigus subtypes and autoantibody profile.^1^ Dsg1 is concentrated in the superficial epidermis, whereas Dsg3 predominates in basal and suprabasal layers, especially mucosa. Accordingly, mucosal-dominant PV is associated with anti-Dsg3 antibodies alone, whereas mucocutaneous PV involves both anti-Dsg1 and anti-Dsg3. These antibodies cause intraepidermal acantholysis and fragile blister formation.

Although the patient later experienced a few small erosions on the back and legs, these remained limited. Her clinical and serologic features with borderline then negative anti-Dsg1 and elevated anti-Dsg3 fit mucosal-dominant PV, according to modified subtype criteria allowing minimal cutaneous involvement in absence of anti-Dsg1.^8^

Anatomically, the vulva and perineum are transition zones between keratinized skin and mucosa. These regions are histologically characterized as compact to intermediate stratum corneum rather than classic basket-weave pattern of typical epithelium.^9^ Given that Dsg1 localizes to superficial layers of fully keratinized epithelium, the distinct stratum and more prominent granular layers of the perineum and interlabial sulcus likely reflect lower Dsg1 reserve and more mucosal-like desmoglein profile where dsg3 predominates.^9^ These features may increase susceptibility to Dsg3-mediated acantholysis.

Serial anti-Dsg3 measurements with titer rising from 91 U/mL to 1508 RU/mL (with RU and U equivalent in ELISA reporting) paralleled onset of increased oral-mucosa involvement highlighting utility of desmoglein assay for monitoring disease activity, especially when biopsy is deferred.

On initial presentation with an isolated plaque and modest anti-Dsg3, negative IIF likely reflected low circulating antibody titers below IIF detection threshold in early or localized PV. Thus, although both ELISA and IIF can aid diagnosis, perilesional DIF (detecting tissue-bound IgG) remains the diagnostic gold standard for prompt intervention, and prevention of complications. A multidisciplinary evaluation is crucial because PV often affects various mucosal sites. With reports indicating up to 40% of cases involving the larynx,^10^ persistent laryngitis reported in this case prompted assessment by an otolaryngologist.

Perineal erosions can be the first and only manifestation of PV for months. Dermatologists, primary care, gynecologists, and urologists should maintain high suspicion and low threshold for pemphigus diagnostic workup, including histopathology, perilesional DIF, or desmoglein ELISA with close follow-up for timely diagnosis and treatment.

## Conflicts of interest

None disclosed.
